# The Biogenic Amine Tyramine and its Receptor (AmTyr1) in Olfactory Neuropils in the Honey Bee (*Apis mellifera*) Brain

**DOI:** 10.3389/fnsys.2017.00077

**Published:** 2017-10-24

**Authors:** Irina T. Sinakevitch, Sasha M. Daskalova, Brian H. Smith

**Affiliations:** ^1^School of Life Sciences, Arizona State University, Tempe, AZ, United States; ^2^Biodesign Center for BioEnergetics, Arizona State University, Tempe, AZ, United States

**Keywords:** biogenic amine receptors, G-protein coupled receptors, tyramine, learning and plasticity, olfactory pathways

## Abstract

This article describes the cellular sources for tyramine and the cellular targets of tyramine via the Tyramine Receptor 1 (AmTyr1) in the olfactory learning and memory neuropils of the honey bee brain. Clusters of approximately 160 tyramine immunoreactive neurons are the source of tyraminergic fibers with small varicosities in the optic lobes, antennal lobes, lateral protocerebrum, mushroom body (calyces and gamma lobes), tritocerebrum and subesophageal ganglion (SEG). Our tyramine mapping study shows that the primary sources of tyramine in the antennal lobe and calyx of the mushroom body are from at least two Ventral Unpaired Median neurons (VUMmd and VUMmx) with cell bodies in the SEG. To reveal AmTyr1 receptors in the brain, we used newly characterized anti-AmTyr1 antibodies. Immunolocalization studies in the antennal lobe with anti-AmTyr1 antibodies showed that the AmTyr1 expression pattern is mostly in the presynaptic sites of olfactory receptor neurons (ORNs). In the mushroom body calyx, anti-AmTyr1 mapped the presynaptic sites of uniglomerular Projection Neurons (PNs) located primarily in the microglomeruli of the lip and basal ring calyx area. Release of tyramine/octopamine from VUM (md and mx) neurons in the antennal lobe and mushroom body calyx would target AmTyr1 expressed on ORN and uniglomerular PN presynaptic terminals. The presynaptic location of AmTyr1, its structural similarity with vertebrate alpha-2 adrenergic receptors, and previous pharmacological evidence suggests that it has an important role in the presynaptic inhibitory control of neurotransmitter release.

## Introduction

The biogenic amines tyramine and octopamine are neuroactive compounds that are involved in a large repertoire of invertebrate behaviors, including locomotion, sensory processing, learning and memory (Roeder, 2005; Scheiner et al., 2006; Lange, 2009). Since Erspamer and Boretti (1951a,b) first described octopamine in the salivary gland of the octopus as having “adrenaline-like” action, many studies have demonstrated the important role octopamine and its biosynthetic precursor tyramine play in invertebrate physiology and behavior. The source of octopamine was mostly allocated to the paracrine cells, the so-called dorsal (ventral) unpaired median (DUM/VUM) neurons, first described by Plotnikova (1969). Due to their location on the midline of the ventral nerve cord and brain, and to their unique morphology, Hoyle (1974) proposed that these octopaminergic neurons are involved in the modulation of many types of behavior.

Tyramine is synthesized from tyrosine by the enzyme tyrosine decarboxylase, and then octopamine is synthesized from tyramine in one step by the action of enzyme tyramine beta-hydroxylase (David and Coulon, 1985). Until recently, tyramine was thought only to be the precursor of octopamine, without playing any other significant role. Studies of tyramine and its receptors in invertebrates clearly indicated that tyramine has sources and functions independent of octopamine (Kutsukake et al., 2000; Roeder et al., 2003; Alkema et al., 2005; Roeder, 2005; Lange, 2009; Bayliss et al., 2013; Scheiner et al., 2014; Ishikawa et al., 2016). In the locust, tyramine is expressed in all neurons that express octopamine as well as in some cells that do not express either the beta-hydroxylase enzyme or octopamine (Kononenko et al., 2009; Homberg et al., 2013). Studies in the fruit fly larval central nervous system also reported the presence of tyramine-containing neurons that are distinct from octopaminergic neurons (Nagaya et al., 2002).

Tyramine and octopamine trigger intracellular signaling pathways by binding with different affinities to a variety of octopamine receptors (OARs) or tyramine receptors (TYRs), most of which are G-protein coupled receptors (GPCRs; Grohmann et al., 2003; Balfanz et al., 2005; Hauser et al., 2006). There are four different classes of invertebrate GPCRs that bind octopamine and/or tyramine (Evans and Maqueira, 2005; Maqueira et al., 2005; Verlinden et al., 2010; Bayliss et al., 2013; Balfanz et al., 2014). Alpha-adrenergic-like OARs (OctαRs/OA1), beta-adrenergic-like OARs (OctβRs/OA2), octopamine/tyramine (Oct/Tyr/TyrR I) receptors, and TyrR II (Verlinden et al., 2010). When it binds with octopamine, AmOA1 releases calcium from cytosolic stores (Grohmann et al., 2003). The OA2 receptor stimulates adenylyl cyclase activity, which leads to an increase of 3,5–cyclic monophosphate (cAMP; Robb et al., 1994; Roeder, 1999; Maqueira et al., 2005; Balfanz et al., 2014). TyrR I (in the honey bee, AmTyr1 or AmTAR1) preferentially binds to tyramine and inhibits adenylyl cyclase activity. TyrR II (in the honey bee AmTAR2) can mediate calcium signaling and/or affect cAMP levels (Blenau et al., 2000; Verlinden et al., 2010; Ohta and Ozoe, 2014; Reim et al., 2017).

AmTyr1 has been cloned and characterized (Blenau et al., 2000; Blenau and Baumann, 2001, 2003). Earlier localization studies using *in situ* hybridization indicated that AmTyr1 is expressed on cell bodies of mushroom body Kenyon cells (KCs) and in the antennal lobe (Mustard et al., 2005). In the cockroach, PeaTyr1 is expressed in abundance in all brain neuropils as well as in peripheral tissues such as the salivary glands (Rotte et al., 2009). A recent study by Reim et al. (2017) characterized the *Apis mellifera* TYR type 2 (AmTAR2). The authors provide evidence that AmTAR2, when heterologously expressed in flpTM cells, exclusively causes an increase in cAMP.

Here we use immunocytochemistry to describe the localization of tyramine and its receptor AmTyr1 in the olfactory networks of the antennal lobe and mushroom bodies. We focused on AmTyr1 because it has been implicated in genetic studies of foraging and reproductive behaviors as well as in olfactory learning in honey bees (Chandra et al., 2010; Wang et al., 2012; Scheiner et al., 2014). The antennal lobe of the honey bee is the anatomical and functional analog of the vertebrate olfactory bulb (Hildebrand and Shepherd, 1997). The antennal lobe consists of an aglomerular neuropil that is surrounded by 160 glomeruli, where each glomerulus participates in coding for a subset of odors. The cortex—the outer rind—of each glomerulus receives olfactory receptor inputs from axons of olfactory receptor neurons (ORNs). Each glomerulus contains dendrites of Projection Neurons (PNs), axons from which then connect the antennal lobe to higher order processing centers—the lateral horn (LH) and mushroom body calyx. The PN dendrites in the core of a glomerulus receive synapses from local neurons (Sinakevitch et al., 2013). The mushroom bodies are higher order olfactory processing centers. They contain intrinsic neurons—the KCs—that have cell bodies packed around the mushroom body calyces. The dendrites of KCs in basal ring and lip of the calyces receive olfactory and gustatory afferents (Strausfeld, 2002). The dendrites of KCs in the collar area of the calyx receive visual afferents. KCs axons make up the peduncle and mushroom body lobes: vertical, medial and γ (Strausfeld, 2002).

Tyramine release in the antennal lobe and mushroom body could modulate this network at several points, but the precise anatomical distribution of tyramine and its receptors has not been analyzed in detail, except for a publication contemporary to ours (Thamm et al., 2017). In our study, we used antibodies against conjugated tyramine to show its immunolocalization, and we compared the distribution of anti-tyramine staining to the distribution of octopamine staining published in earlier work (Kreissl et al., 1994; Sinakevitch et al., 2005). We also generated and characterized antibodies against AmTyr1 protein and used them to identify the distribution of the AmTyr1 receptor on neurons that are critical components of the olfactory circuitry. Our study shows how tyramine via AmTyr1 is poised to modulate odor processing at different points in the honey bee brain.

## Materials and Methods

### Animals

Honey bees (*Apis mellifera* L.) were adult New World Carniolan foragers of unknown age obtained from colonies maintained at Arizona State University. The bees were collected at the entrance of the hive when they returned from the field with pollen, usually in the afternoon.

### Anti-Tyramine Staining

Tyramine antiserum (AB124; EMD Millipore) was raised in rabbits using p-tyramine conjugated to N-alpha-acetyl-L-lysine-N-methylamide using glutaraldehyde (Geffard et al., 1984b). This antiserum has been used in the locust and fruit fly to describe tyramine-like immunoreactivity in the brain (Nagaya et al., 2002; Kononenko et al., 2009; Homberg et al., 2013). Antiserum specificity of immunostaining was tested using tyramine/octopamine conjugated to bovine serum albumin (BSA). The conjugates were prepared as described elsewhere (Geffard et al., 1984a; Mons and Geffard, 1987). Brains were dissected out of the head in fixative containing 3% glutaraldehyde (Electron Microscopy Sciences (EMS), Hatfield, PA, USA) in 0.1 M cacodylate buffer (EMS, pH 7.0) with 1% sodium metabisulfite (SMB, Sigma-Aldrich, St.Louis, MO, USA). Each dissected brain was then transferred into 1 ml of fresh fixative and left overnight at 4°C. To saturate double bonds, after fixation the brains were treated with 0.5% sodium borohydride (NaBH4; Sigma-Aldrich) in 0.05 M Tris-HCl buffer containing 0.45% SMB (Tris-HCl-SMB; Sigma), pH 7.4 for 20 min. After washing with Tris-HCl-SMB buffer (4 × 10 min), the brains were embedded in 8% agarose (low melted point A0169, Sigma-Aldrich) in water. Brain sections (70 μm) were made with a Vibratome Leica 1000S (Leica Biosystem, Germany).

Brain sections were washed (6 × 20 min) in Tris-HCl-SMB buffer containing 0.5% Triton-X100 (TX) and were incubated with 1% normal donkey serum (Jackson ImmunoResearch Laboratories) in Tris-HCl-TX for 15 min. Then, tyramine antiserum was added to a final dilution of 1:500 to each brain and left for 48 h. After washing in 0.05 M Tris-HCl-TX, pH 7.5 (6 × 20 min), F(ab’)2 fragments of donkey anti-rabbit IgG conjugated to Cy3 (Jackson ImmunoResearch Laboratories, diluted 1:250 in Tris-HCl-TX) were applied as the secondary antibody overnight. All incubations were at room temperature. After final washing in 0.05 M Tris-HCl pH 7.4 (6 × 20 min) sections were mounted on slides in 80% glycerol in phosphate-buffered saline (PBS) mix.

**Figure 1:**
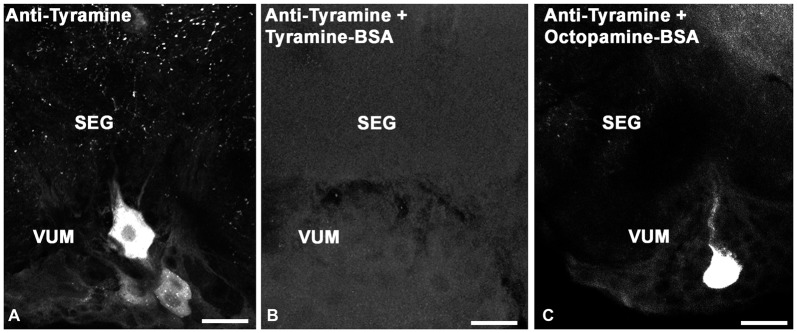
Controls for immunolabeling with tyramine antiserum. We used three sections of the subesophageal ganglion (SEG) that contain ventral median group cells (VUM). The sections were labeled with one of the following: **(A)** tyramine antiserum; **(B)** tyramine antiserum pre-incubated with conjugated tyramine-bovine serum albumin (BSA); **(C)** tyramine antiserum pre-incubated with octopamine-BSA. Scale bar: 25 μm.

To test the specificity of tyramine immunostaining, working dilutions of the anti-tyramine antibodies were preincubated overnight without and with tyramine conjugated to BSA, (10^−4^ M concentration of tyramine in the conjugate, Figures 1A,B). After preincubation of the primary antiserum with tyramine-G-BSA, anti-tyramine staining was absent (Figure 1B). However, after preincubation of the antibodies with octopamine-G-BSA (10^−4^ M concentration of octopamine; Figure 1C), immunolabeling in the cell body and fine processes was present. Therefore, the working dilution of the tyramine antiserum specifically recognizes tyramine in cell bodies and their processes in fixed honey bee brain sections. Twenty bee brains were processed and analyzed in our studies, eight brains that were fixed and processed on the same days were used to count cell bodies (Table 1).

**Table 1:** The number of tyramine-like immunoreactive neurons in clusters of cells in the brain and subesophageal ganglion of *Apis mellifera*^1,2^.

	Bee *N* = 8 No. of cells on each side	Neuropils supplied in Apis
Cluster G0	3 (10)	NCC1
Cluster G0b	None	
Cluster G1	None	
Cluster G2a	6 (25)	EB, FB, M Pro (circum Ped, V Lo, α lobe ?
Cluster G2b	5 (10)	V-M Pro
Cluster G3a	6 (25) + 3 (15)	L Pro, Op Lo, Oc N, PB, FB, γ lobe?
Cluster G3b	2 (15)	L Pro
Cluster G4a	2 (10)	PB and EB
Cluster G4b	16 (10) + 1 (25)	PB, EB, D-M Pro
Cluster G4c	2 (15)	L Pro
Cluster G4d	4 (8)	
Cluster G5a	None	
Cluster G5b	5 (25) + 5(8)	L Pro, M Pro, Ant Mech, Ant nerve, sub CB, OpLo
Cluster G6a	3 (25)	Deu, Trito, Ant N? SEG
Cluster G6b	3 (10)	SEG
Ventral unpaired median		
(VUM) neurons mandibular	8^3^ (25)	SEG Md Neuromere, Trito L Ho, Ca, Ant Lo, Ant Nerve, NCC1?
VUM neurons maxillary	8^3^ (25)	SEG, L Ho, Ca, Ant Lo, L Pro, SEG, Mx nerve
Ventral paired median (VPM) neurons	2^3^ (25)	L Pro, M Pro, NCC1?
VUM neurons labial	8^3^ (25)	SEG Labial Neuromere, NCC1
DUM neurons labial	2^3^ (25)	?

*^1^The terminology used for cell groups is the same as in Sinakevitch et al. (2005). ^2^The approximate sizes of cell bodies are shown in parenthesis in μm. ^3^Total number of cell bodies on midline of SEG. NCC, nervii corpora cardiacii; FB, fan-shaped body; EB, ellipsoid body; PB, protocerebral bridge; D, L, M, or V Pro, dorsal, lateral, medial, or ventral protocerebrum; Ant Lo, antennal lobe; Op Lo; SEG, subesophageal ganglion; Trito, tritocerebrum, Deu, deutocerebrum (other than Ant Lo and Ant Mech); Ant Mech, antennal mechanosensory neuropil; sub CB, neuropil beneath ellipsoid and fan-shaped body; circum Ped, protocerebral neuropil surrounding pedunculus and vertical lobe (V Lo); α and γ division of mushroom body vertical lobe; L Ho, lateral horn of protocereberum; Ca, calyx; Oc N, ocellar nerve*.

### Immunocytochemistry with Anti-Synapsin Antibody

Immunocytochemistry with mouse monoclonal anti-synapsin antibody (SYNORF1; clone 3C11, Developmental Studies Hybridoma Bank, The University of Iowa) was used to label the synaptic neuropil in brain whole-mounts according to the protocol of Brandt et al. (2005). Brains (*n* = 3) were fixed in 4% paraformaldehyde in PBS overnight, washed in PBS containing 1% Triton-X (PBS-TX), preincubated 1 h with normal donkey serum, and then the anti-synapsin antibody 1:1000 was applied for 3 days at room temperature under gentle shaking. After six washes of 1 h each with PBS-TX, the brains were incubated with the secondary antibody (F(ab’)2 fragments of donkey anti-mouse conjugated to Alexa 488, Jackson ImmunoResearch Laboratories) diluted 1:250 in PBS-TX for 2 days at room temperature. After final washing in PBS, the whole-mounts were dehydrated, cleared and embedded in methyl salicylate. The whole-mount brains were embedded in methyl salicylate for observation (*n* = 3). To illustrate the location of the anti-tyramine staining cell bodies, we used the consecutive protocol staining technique on sections. Three brains that were sectioned and labeled with anti-tyramine were post-fixed for 20 min with 4% paraformaldehyde, and then brain sections were processed for anti-synapsin immunostaining as described above to obtain tyramine immunoreactive cell bodies in the sections with the labeled synaptic structure of neuropil (*n* = 3).

### Three Dimensional (3D) Model of the AmTyr1 Receptor Structure

The structural model of AmTyr1 (NCBI Reference Sequence: NP_001011594.1) was generated by I-TASSER (Zhang, 2008; Roy et al., 2010; Yang and Zhang, 2015; Yang et al., 2015) based on the top 10 proteins from the PDB that have the closest structural similarity. Besides the threading-based restraints, no additional external restraints were specified. Molecular graphics and localization of the antigenic peptides were performed with the UCSF Chimera package (Pettersen et al., 2004) developed by the Resource for Biocomputing, Visualization and Informatics at the University of California, San Francisco.

### Anti-AmTyr1 Receptor Antibody

#### Design of Conjugated Peptides and Antibody Production

**Figure 2:**
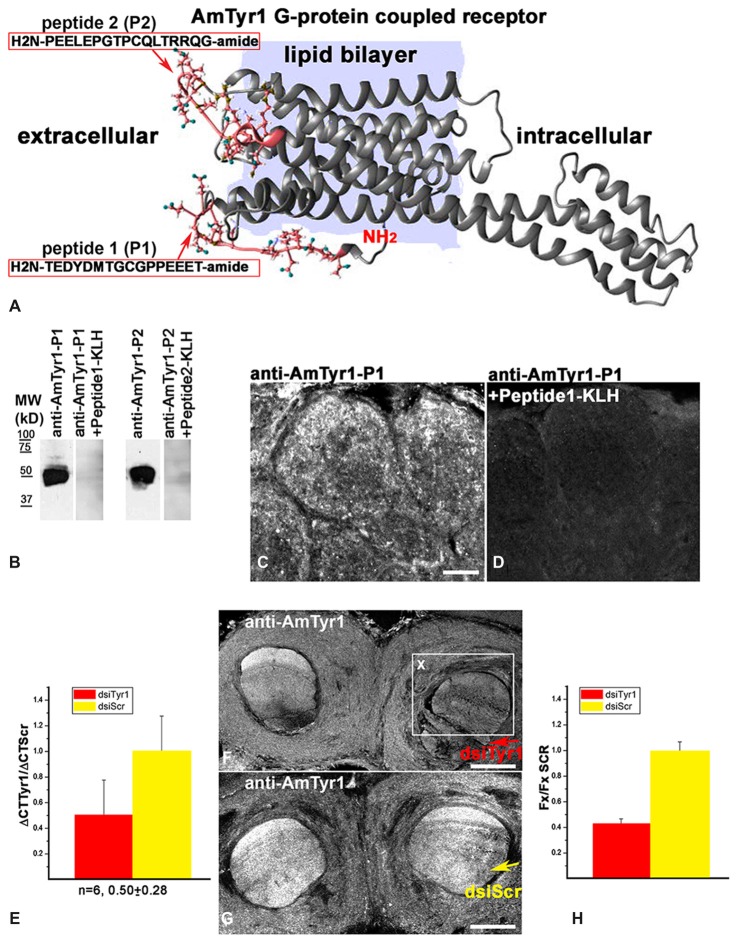
Characterization of anti-AmTyr1 antibodies. **(A)** Predicted structural model of the *Apis mellifera* tyramine receptor (NP_001011594.1). Peptides (P1, P2) from the N-terminal region and the loop between helices 4 and 5 used to generate the antibodies are represented by their amino acid sequence (pink). **(B)** Affinity purified anti-AmTyr1 antibodies against peptide-1 and peptide-2 were tested in western analyses. The relative positions of molecular weight (MW) standards in kDa are indicated. The affinity purified anti-AmTyr1-P1 and anti-AmTyr1-P2 each revealed a band corresponding to an approximate molecular weight of 45 KDa. Preincubation of the anti-AmTyr1 antibodies separately with corresponding peptide conjugates abolished the band. **(C)** Anti-AmTyr1 immunostainings in the antennal lobe revealed processes in the cortex of glomeruli on frontal section of the brain. **(D)** In the next consecutive section, staining in glomeruli was not present when the anti-AmTYR1 antibodies were pre-incubated with Keyhole Limpet Hemocyanin (KLH)-conjugated peptide-1 before immunostaining. **(E)** Expression of *AmTyr1*gene in brains injected with 70 nl of 100 μM dsiAmTyr1 RNA or dsiScramble 14 h after treatments. *AmActin* was used as a reference gene. The relative gene expression was calculated using the 2^−ΔΔCt^ method. The data are expressed as mean ± SE. **(F)** Anti-AmTyr1 staining in the brain section 18 h after injection dsiAmTyr1 RNA **(G)** and dsiScr. Arrows in **(F,G)** indicate injections sites in the frontal sections of the bee brains.** (H)** Quantification of the average fluorescence intensity value in the box X (Fx), outlined in F in the raw images of brains that were injected with dsiTyr1 and dsiScr. Images were collected with a confocal fluorescent microscope with the same gain settings and intensity level. Relative intensity level of fluorescence dropped to 42 ± 5% (mean ± SE) in the dsiTyr1 injected brains compared to dsiScr brains in the local area of the injections. Scale bar: **C,D** = 10 μm, **F,G** = 100 μm.

Anti-AmTyr1 receptor antibodies were produced in two rabbits immunized with two peptides from the N-terminus of AmTyr1 (H_2_N-TEDYDMTGCGPPEEET-amid (peptide-1, P1) and H_2_N-PEELEPGTPCQLTRRQG-amide (peptide-2, P2) conjugated to Keyhole limpet hemocyanin (KLH; Figure 2A). After four immunizations, serum from two rabbits was collected and affinity purified. All these procedures were performed by 21st Century Biochemical Incorporation (Marlboro, MA, USA).

#### Western Blot

The affinity purified anti-AmTyr1 antibody raised against peptide-1 and peptide-2 were further characterized by western blot (Figure 2B). The brains were dissected and processed for membrane protein extraction. Each individual brain was homogenized in 100 μl of lysis buffer (120 mM Tris-HCl, 2% sodium dodecyl sulfate (SDS), 5% glycerol, 0.2 mM dithiothreitol, 1% Triton X100 containing 5 μg/ml of each protease inhibitors PMSF (Phenylmethylsulphonylfluoride), Aprotinin, Benzamidine (all from Sigma-Aldrich) pH 6.8). Homogenates were centrifuged at 12,000 *g* for 20 min at 4°C. Then 30 μl (1/3 of a bee brain) of the supernatant was added with 6 ×Laemmli buffer and loaded on a 7.5% SDS-polyacrylamide Tris-glycine gel to separate the proteins. Proteins were transferred onto nitrocellulose membranes (Bio-Rad Laboratories) in transfer buffer (25 mM Tris-HCl, 192 mM glycine, 15% methanol) at 0.45 amp for 1 h 30 min at 4°C. Then the membranes were blocked for 1 h in PBS containing 0.1% Tween-20 (PBS-Tw) and 5% low fat powdered milk. Then they were incubated with anti-AmTyr1 antibody raised against conjugate of peptide-1 and peptide-2, each on separate membrane at 1:1000 in PBS-Tw plus 5% milk for 4 h at room temperature. Following four 15 min washes in PBS-Tw with 5% milk, membranes were incubated with anti-rabbit IgG HRP-conjugated secondary antibodies (Rockland Inc.) at 1:10,000 in PBS-Tw with 5% milk for 2 h. Membranes were washed four times in PBS-Tw and developed using chemiluminescence as described by the manufacturer (Immobilon Western Chemiluminescent HRP Substrate; Millipore Corporation). The preadsorption control was done with the corresponding peptide-conjugated to KLH with each anti-AmTyr1 antibodies where the peptide concentration was approximately 10^−5^ M. In these procedures, anti-AmTyr1 + 1 × 10^−5^ M peptide-KLH were incubated for 2 h at 37°C with gentle shaking. Then after centrifugation for 10 min at 10,000 *g* at 4°C, the supernatant was applied on the membrane and processed as described above. This treatment abolished staining on the membrane for both antibodies.

### Anti-AmTyr1 Staining

For all immunostaining on bee brain sections, we used the anti-AmTyr1 antibodies that were raised against the peptide-1, and the protocol for testing their immunostaining specificity on bee brain sections is described below.

Honey bee forager brains were removed from the head capsule under fixative containing 4% paraformaldehyde in PBS, then each brain was placed in one milliliter of the same fixative overnight at 4°C. The next morning, brains were washed in PBS and embedded in 8% agarose. Each agarose block was sectioned (70 μm) and processed for immunostaining. First, brain sections were preincubated with 1% normal donkey serum and then anti-AmTyr1 (affinity purified polyclonal antibodies raised against the peptide-1 in rabbit) at 1:500 dilution in the PBS-0.5%TX solution was added to each brain. To visualize the staining, F(ab)’2 fragments of donkey anti-rabbit IgG conjugated to Cy3 were used at a dilution of 1:500. After each step, there were at least six washes of 20 min each in PBS-TX (6 × 20 min). PBS only was used in the final wash before embedding brain sections on a glass slide. Twenty brains were processed with anti-AmTyr1 antibodies in these experiments.

#### Characterization Specificity of Immunostaining of anti-AmTyr1 (Affinity Purified Antibodies Raised Against Peptide-1) on Bee Brain Sections

To examine the specificity of immunostaining of the anti-AmTyr1 antibodies, sections were incubated with the secondary antibody in the absence of primary antibodies (not shown) or immunoassay with working dilution of anti-AmTyr1 (Figure 2C) or anti-AmTyr1 antibody that had been preincubated with KLH conjugated to AmTyr1 peptide-1 via glutaraldehyde (Figure 2D). In these procedures, anti-AmTyr1 and anti-AmTyr1 + 2 × 10^−4^ M peptide-1-KLH were incubated for 2 h at 37°C with gentle shaking. Then after centrifugation for 10 min at 10,000 *g* at 4°C, the supernatant of each solution was applied on two consecutive sections and processed as described above. All procedures were performed at room temperature unless otherwise noted. Images of sections treated with anti-AmTyr1 antibody preincubated with conjugated peptide were collected at the same level of gain and intensity.

#### Control for Immunostaining after Knockdown of AmTyr1

To demonstrate that anti-AmTyr1 antibodies specifically recognized the receptor, we used a Dicer-substrate small interfering (dsi) RNA of the *AmTyr1* receptor (NCBI Reference Sequence: NM_001011594.1) to knock down levels of the *AmTyr1* mRNA receptor in the brain (Figure 2E). We used the mixture of three dsiAmTyr1 designed by the tool in IDT technology (Table 2), and as a control we used scrambled dsiScr. Seventy nanoliters (nL) of 100 μM mixture of dsiAmTyr1 or dsiScr was injected using a picospritzer into the mushroom body lobe on one side (six bees for each group), and then the brain without optic lobes was dissected and homogenized in 1 ml of TRIzol (Invitrogen) 13–15 h after injections. Then the total mRNA from each injected bee brain part was extracted separately using the manufacturer’s protocol for TRIzol method (Invitrogen). Contaminating genomic DNA was removed using DNA-free™ kit (Ambion, AM1906). RNA quantity and purity was evaluated using a NanoDrop (NanoDrop 2000). Expression of AmTyr1 was quantified using QuantiFAST SYBR Green RT-PCR kit (QIAGEN, 204156) on Applied Biosystem 7500 cycler with the exact protocol provided by the 96-well kit. We used 20–40 ng of RNA per well. The primers for quantitative real-time PCR assays were characterized and described in Wang et al. (2012): AmTyr1_F 5′-GTTCGTCGTATGCTGGTTGC-3′, AmTyr1_R 5′-GTAGATGAGCGGGTTGAGGG-3′ and for reference gene AmActin_F 5′-TGCCAACACTGTCCTTTCTG-3′ AmActin_R 5′-AGAATTGACCCACCAATCCA-3′. The relative gene expression was calculated using the 2^−ΔΔCt^ method.

**Table 2:** Nucleotide sequences of sense and antisense strands of control DsiSCR and AmTyr1 DsiRNA.

DsiRNA	Sequences
dsiScr	5′-GAGUCCUAAGUUAACCAAGUCACAGCA-3
	3′-CUCAGGAUUCAAUUGGUUCAGUGUCGU-5′
dsiTyr1_N	5′-AGCGUGACGUUGGAUUGACGAGAGC-3′
	3′-CCUCGCACUGCAACCUAACUGCUCUCG-5′
dsiTyr1_T1	5′-CCUGUGCAAAUUGUGGCUAACCUGC-3′
	3′-GUGGACACGUUUAACACCGAUUGGACG-5′
dsiTyr_C	5′-CAACGCUUGUUUAUUGCAUCUAUCG-3′
	3′-CCGUUGCGAACAAAUAACGUAGAUAGC-5′

To test immunostaining of the AmTyr1 protein, five dsiAmTyr1 injected brains and five control brains injected with dsiScr were dissected and fixed 18–24 h after injections, then processed for anti-AmTyr1 immunostaining as described in section anti-AmTyr1 staining above (Figures 2F–H). To estimate the reduction of protein in fixed brain tissue we used the original raw images of brains injected with dsiTyr1 and dsiScr collected at the same gain. The area of interest X was drawn on each section in the place where dsiRNA was injected (Box X in Figure 2F). The average fluorescence intensity value (Fx) was estimated in Adobe Photoshop CC 2015 on each sections on the injected side of the brain. Then statistical analyses and the graph were done in Origin 6.1 software.

### Triple Staining with anti-AmTyr1, Anti-Synapsin and Neurobiotin Labeled Neurons

To identify cell types that express AmTyr1 in the antennal lobe, we labeled ORN and PN terminals by injecting neurobiotin into the antenna and antennal lobe. For both procedures, each bee was cooled and placed into a plastic restraining harness, and the head was immobilized with low melting point wax. Then each antenna was gently immobilized with ecosaine, the isomeric hydrocarbons obtained from paraffin wax (Aldrich).

To label ORN endings in the glomeruli, neurobiotin was injected into the antenna, where the ORN axons take up the tracer, which then undergoes anterograde transport to the axon terminals in glomeruli. For neurobiotin injection into the antenna, a small hole was cut in the scape at the base of the antenna, and 50–70 nL of 2% neurobiotin (weight/volume in water) was injected using a picospritzer. To label ORN cell bodies in the antenna, neurobiotin was injected into the antennal lobe from where the tracer was transported retrogradely to the cell body and dendritic fibers in the sensory receptor pocket. 4′,6-diamidino-2-pheylindole (DAPI) was used as a fluorescent marker of cell nuclei in the antenna. Five bees were processed to trace the ORNs and co-labeled with anti-AmTyr1 and anti-synapsin.

To label the PN axon terminals we injected neurobiotin into the antennal lobe, where the PNs take up the tracer and anterogradely transport it to the mushroom body calyx, where axons form the synapses in the KC dendritic field. For this procedure, a small window was cut in the head capsule allowing access for neurobiotin injection into each antennal lobe. The bee then was detached from the holder and placed in a small wooden box with available food (1.5 M sucrose and pollen) and a humidified environment. The bees were sacrificed the next day 16–20 h after dye injections. Brains were dissected and fixed as described above for anti-AmTyr1 staining. Five bees were processed to trace the PNs and co-labeled with anti-AmTyr1 and anti-synapsin.

To label synaptic neuropil, the anti-synapsin antibody (dilution 1:1000) was added together with anti-AmTyr1 (1:500) overnight. Then sections were washed, and secondary antibodies F(ab’)2 fragments donkey anti-rabbit IgG conjugated to Cy3 (1:250) and F(ab’)2 fragments donkey anti-mouse IgG conjugated to 488 (1:250) were added to the sections together with streptavidin conjugated to Cy5 (Jackson ImmunoResearch Laboratories, 1:250) to reveal anti-AmTyr1, anti-synapsin and neurobiotin respectively in the brain sections. Preparations were then thoroughly washed in PBS and embedded in 80% glycerol. To control the specificity of the secondary antibodies, all secondaries were incubated with sections that had only one of the primary antibodies. The staining did not show any cross-reaction between the secondary antibodies and Streptavidin-Cy5. Streptavidin-Cy5 did not interact with any structure in the absence of the neurobiotin in the bee brain.

### Confocal Microscopy

**Figure 3:**
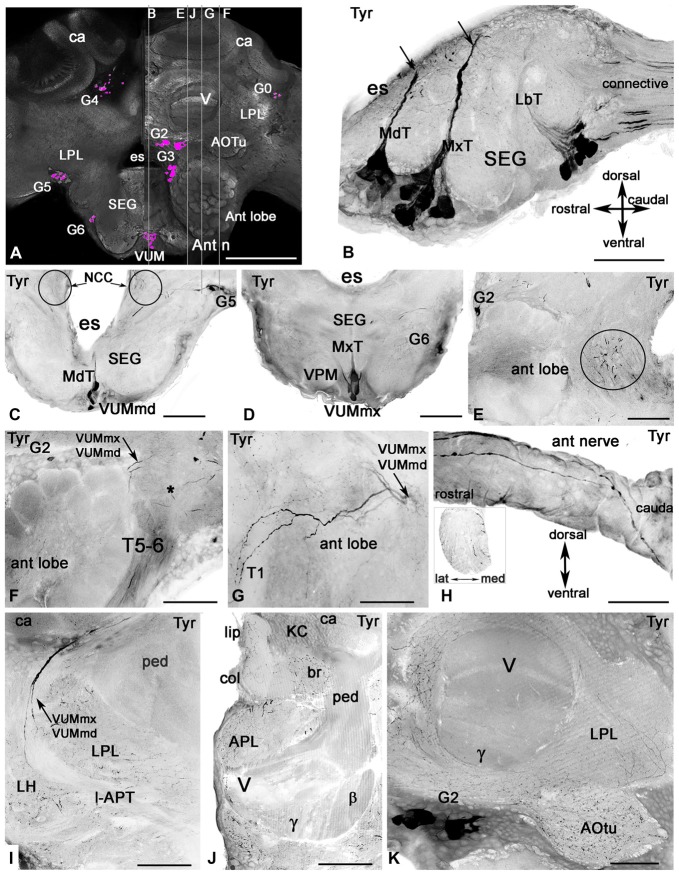
Tyramine-like immunoreactivity in the honey bee brain using inverted fluorescence images. **(A)** Schematic representation of the brain (frontal view) with the groups of cell bodies labeled with anti-tyramine antibodies. The left and right halves of the schematic demonstrate the caudal and rostral planes of the brain. The tyramine containing cell groups (magenta) are G2-G6 and VUM. The plane of the sagittal sections on corresponding images **(B,E,F,G,J)** is indicated by the vertical lines. **(B)** The sagittal section through the SEG with anti-tyramine labeled groups of median neurons in mandibular (Md), maxillary (Mx) and labial (Lb) neuromeres with their primary neurites in corresponding Md (MdT), Mx (MxT) and Lb (LbT) tracts. **(C,D)** Frontal sections of SEG made via Md **(C)** and Mx **(D)** neuromeres respectively, show corresponding frontal view of VUMmd **(C)** and VUMmx **(D)** and ventral paired median (VPM) neurons. The VUM neurons send their primary neurites to the corresponding tracts, and the secondary neurites branch in the deutocerebrum (circle in **C,E**). **(F,G)** Two tyramine immunoreactive axons from VUM neurons, one from MdN and one from MxN, innervate the antennal lobe (sagittal sections, front on the left) and give rise to ramifications in glomeruli and aglomerular neuropils of the antennal lobe **(G)**. Asterisk in **(F)** indicates dorsal lobe. **(G)** The tyramine immunoreactivity in tract T5-T6 is from unidentified neurons in the tritocerebrum and SEG. **(H)** These unidentified tyramine immunoreactive neurons enter into the antennal nerve and are running along the top of the antennal nerve in the sagittal view and inside of the nerve (frontal view, insert). **(I)** The secondary neurites from tyramine immunoreactive VUMmd and mx enter in the lateral antenna-protocerebral tract (l-APT) and innervate the lateral horn (LH) and mushroom body calyx (ca). **(J)** In the mushroom body calyx, they innervate the basal ring (br) and lip areas, which receive olfactory afferents from the antennal lobe. The mushroom body pedunculus (ped) and lobe are almost free from tyramine immunoreactive innervation **(I,J,K)** except for a few branches in the γ lobe **(K)** that might originate from LPM neurons from SEG. The arrow in **(C)** indicates tyramine immunoreactive fibers running alongside of the esophageal (es) to the corpora cardiaca nerve (NCC). Ant lobe, antennal lobe; ca, calyx of mushroom body; SEG, subesophageal ganglion; VUM, ventral unpaired median neurons; Ant n, antennal nerve; LPL, lateral protocerbral lobe; APL, anterior protocerebral lobe; AOTu, anterior optic tubercule; V, γ, β, vertical lobe of mushroom bodies, KC, Kenyon cell bodies. Scale bar: **A** = 250 μm, **B** = 50 μm, **C–K** = 100 μm.

Data were collected on a Leica SP5 confocal laser scanning microscope (Leica, Bensheim, Germany) using a Leica HCX PLAPO CS 40_oil-immersion objective (numerical aperture: 1.25) with appropriate laser and filter combinations. Stacks of optical sections at 1 μm spacing were processed using Leica software (1024 × 1024 pixel resolution) either as a single slice or flattened confocal stacks (maximum intensity projections). Size, resolution, contrast, and brightness of final images were adjusted with Adobe Photoshop software. To generate the general view of a tyramine-containing cell in the whole brain we used whole-mount immunocytochemistry with anti-synapsin antibodies. Serial sections were made at 1 μm using a Leica ×10 objective and reconstructed to create 3D images of the brain using AMIRA software (FEI visualization science group). Then the agarose brain sections labeled with both anti-tyramine and anti-synapsin were compared with digital serial sections of the whole-mount brain, and cell bodies were manually added in the appropriate serial digital layer to create the image of tyramine cell body distribution in the whole-mount brain (Figure 3A).

## Results

### Antibodies and Immunolabeling

The tyramine antiserum used in our studies specifically recognized tyramine, as shown through immunostaining pre-absorption tests (Figures 1A–C). Anti-tyramine immunostainings were repeatable across animals, revealing cell bodies, neurites and varicosities of neurons. The intensity of anti-tyramine staining in the cell bodies was variable, as illustrated in Figure 1A for ventral unpaired neurons: staining ranged from very bright to low intensities. After pre-incubation of working dilutions of anti-tyramine antibodies with tyramine-G-BSA, specific anti-tyramine labeling was abolished, as shown in Figure 1B for SEG frontal sections. When the same working dilution of anti-tyramine antibodies was preincubated with octopamine-G-BSA, the tyramine immunoreactive staining in the cell bodies and processes was still present (Figure 1C). In total, we treated 20 brains with anti-tyramine antibodies, and cell counts in Table 1 were based on eight of the brains.

We created a 3D model of the AmTyr1 receptor protein to show localization of the peptides used for immunization (Figure 2A). We used I-TASSER that employs composite approaches of threading, structural refinement, and ab initio modeling to generate a 3D model of the full-length AmTyr1 receptor. The best predicted model had a C (confidence)-score of −0.89, and the estimated TM (template modeling)-score and RMSD (root mean square deviation) of 0.60 ± 0.14 Å and 8.8 ± 4.6 Å, respectively. Generally, values of the C-score (typically, in the range of [−5, 2]) and the TM-score (range of [0, 1]) higher than −1.5 and 0.5, respectively, are indicative of correct global fold and main chain topology. RMSD is more sensitive to local errors, and it is not unusual to have big values for proteins of considerable length. In the light of the above, the generated model of the AmTyr1 receptor appears to be of reasonably high accuracy/quality.

The AmTyr1 receptor protein has seven transmembrane domains with a large intracellular loop between helixes 5 and 6 and a short C-terminus. The peptides used for immunizations are located extracellularly in the N-terminus and between helixes 4 and 5 (Figure 2A). After four immunizations, the AmTyr1 antisera were affinity purified and the anti-AmTyr1 antibodies were further characterized. First, we used western blotting to analyze anti-AmTyr1 antibodies for both peptides separately (Figure 2B). We observed one large band in the Western blot of brain homogenate proteins, and it corresponded to the predicted weight of the TYR protein 45 kD (Figure 2B) for both antibodies. Moreover, pre-incubation of anti-AmTyr1-peptide-1 and anti-AmTyr1 peptide-2 with corresponding peptide conjugated to KLH abolished the immunolabeling on Western blot (Figure 2B). In all our AmTyr1 receptor immunolabeling on bee brain sections, we used only the antibody raised against H_2_N-TEDYDMTGCGPPEEET-amid (peptide-1, P1). Therefore, we used only this antibody in tests for specificity of immunolabeling.

Control immunolabeling on brain sections revealed that anti-AmTyr1 was strongly expressed in the cortex of glomeruli (Figure 2C), and staining was absent after pre-incubation of anti-AmTyr1 with peptide conjugated to carrier protein KLH (Figure 2D). Furthermore, knockdown experiments using dsiTyr1 RNA injections in an amount that reduced gene expression by approximately 50% (Figure 2E) significantly reduced labeling by anti-AmTyr1 antibodies (Figure 2F). In contrast, areas where the scrambled construct (dsiScr) was injected in the same amount as dsiTyr1 RNA failed to reduce labeling (Figure 2G). The average level of fluorescence intensity dropped to 42% in the area close to the dsiTyr1 injection site compared to the same area around the dsiSCR injection site (illustrated in Figure 2H). Figures 2F,G illustrate the frontal sections of the bee brains through the mushroom body vertical lobes embedded into the anterior protocerebrum. From all of these experiments, we conclude that the anti-AmTyr1 antibodies from rabbit specifically recognize the AmTyr1 receptor protein in Western blots and fixed brain sections.

### Distribution of Tyramine Immunoreactive Cell Bodies and Processes in the Honey Bee Brain and SEG

The tyramine antiserum labeled clusters of cells in the brain (Figure 3A) and on the midline of the SEG (Figures 3A–D). Tyramine containing processes are illustrated in the antennal lobe (Figures 3E–G) and antennal nerve (Figure 3H), the lateral protocerebral lobe, the LH, the mushroom body calyces and lobes (Figures 3I–K) and in the anterior optic tubercule (AOTu; Figure 3K).

The major tyramine containing fibers in the neuropils of trito-, deuto- and proto-cerebral ganglia arose from the ventral neurons located in the midline of SEG (Figures 3A–D). These anti-tyramine labeled median neurons were classified based on the position of their primary neurites in tracts within the three SEG segments corresponding to the mandibular (Md, Figures 3B,C), maxillary (Mx, Figures 3B,D) and labial (Lb, Figure 3B) neuromeres, respectively. These neuromeres receive sensory projections from the nerves of the mouthparts and via the mandibular, maxillary and labial nerves supply muscles of the mouthparts involved in the proboscis-extension reflex (Rehder, 1988; Schröter et al., 2007). The SEG is also the relay station for information in descending and ascending neurons. We focused our study on median ventral neurons from the mandibular and maxillary neuromeres. In particular, we focused on the tyraminergic/octopaminergic ventral unpaired median (VUM) neurons (VUMmx and VUMmd) that send their symmetrical secondary neurites in each part of the brain to innervate the antennal lobe (Figures 3E–G), LH (Figure 3I) and mushroom body calyx (Figures 3I,J).

The group of anti-tyramine immunostained ventral neurons in the labial neuromere (Figure 3B) contains at least two dorsal unpaired neurons (not shown in this illustration). The secondary neurites from some of these neurons branch in the SEG, and there are at least two tyramine containing axons in the labial nerves. The DUM/VUM neurons from the labial neuromere innervate the labial nerves, the labial neuropil of the SEG and the corpora cardiaca (Figures 3B,C, Table 1). There were also tyramine containing axons in the connectives between the SEG and prothoracic ganglion (Figure 3B).

The mandibular and maxillary neuromeres each contain eight anti-tyramine immunoreactive neurons that have a laterally symmetrical morphology in the SEG and branch in the deuto- and proto-cerebrum, the antennal nerve and the lateral nerves (Figures 3E–H). At least two anti-tyramine staining branches are present in each (mandibular and maxillary) lateral nerve on each side of the SEG. The anti-tyramine labeled secondary neurites from some VUMmx and VUMmd neurons travel alongside the esophageal foramen (circle in Figures 3C,E) and run through the dorsal lobe and enter to the antennal tracts T5-T6. There were at least five tyramine containing axons in the ventral antennal nerve (Figure 3F and insert in Figure 3H). The antennal nerve also has large anti-tyramine positive varicose fibers on the surface (Figure 3H).

In our study we were particularly interested in VUMmd1 and VUMmx1 (Figures 3C,D), because of their importance for octopamine-driven behavioral conditioning (Hammer, 1993; Farooqui et al., 2003). They give rise to primary neurites in the corresponding MdT and MxT tracts and then to secondary neurites in the deutocerebrum caudal to the antennal lobe, as illustrated in the frontal section of the SEG (secondary neurites are circled in Figure 3C) and in the sagittal section through the left antennal lobe (Figure 3E). These two VUM neurons send branches into the antennal lobe (Figures 3F,G). In both Figures 3F,G, the anti-tyramine immunolabeled sagittal sections through the antennal lobe illustrate two tyraminergic branches from VUMx1 and VUMd1 (arrows). In Figure 3G, the sections made through the antennal T1 tracts show that each branch from VUMmx1 and VUMmd1 gives rise to additional branches that innervate glomeruli on the dorsal and ventral side of the antennal lobe (Figure 3G). The anti-tyramine labeled processes in each glomerulus have varicosities that could be release points for tyramine not only in glomeruli but also in the aglomerular neuropil.

The secondary neurites from VUMmx1 and VUMmd1 run through the lateral antenno-protocerebral tract (l-APT) (Figure 3I) and give rise to very fine branches in the LH and the calyx of the mushroom body with highest distribution in the lip and basal ring of mushroom body calyx (Figure 3J). These neurons arise from VUMmd1 and VUMmx1 and are identical to octopaminergic neurons in the same neuromere described in earlier studies (Schröter et al., 2007). That conclusion is based on our study with anti-tyramine and anti-octopamine labeling on the sections. No additional axons were labeled in the antennal lobe during these procedures [these data are not illustrated here].

The pedunculus and lobe of the mushroom body have little labeling with anti-tyramine staining, except for a few fine branches that were distinguishable in the gamma and vertical lobes (Figures 3I–K). In contrast, all protocerebral neuropils surrounding the mushroom bodies and lobes contain abundant tyramine containing varicosities (Figures 3I–K). Tyramine immunostaining in the mushroom body calyx has its origin from the VUMmx and VUMmd neurons. However, the mushroom body lobes are scarcely labeled with tyramine immunoreactivity, and staining in the gamma lobe originates from the lateral paired ventral cells (VPM, Figure 3D) as well from unidentified neurons in group G2. The tyraminergic neurons from group G3 also innervate the posterior protocerebrum and optic lobes. The tyramine containing neurons from group G4 innervate the protocerbral bridge and central complex (Figure 1 for cell bodies group location and Table 1). The terminology used for tyramine-containing cell groups is the same that is described in Sinakevitch et al. (2005).

### Distribution of AmTyr1 Receptor in the Antennal Lobes and Mushroom Bodies

**Figure 4:**
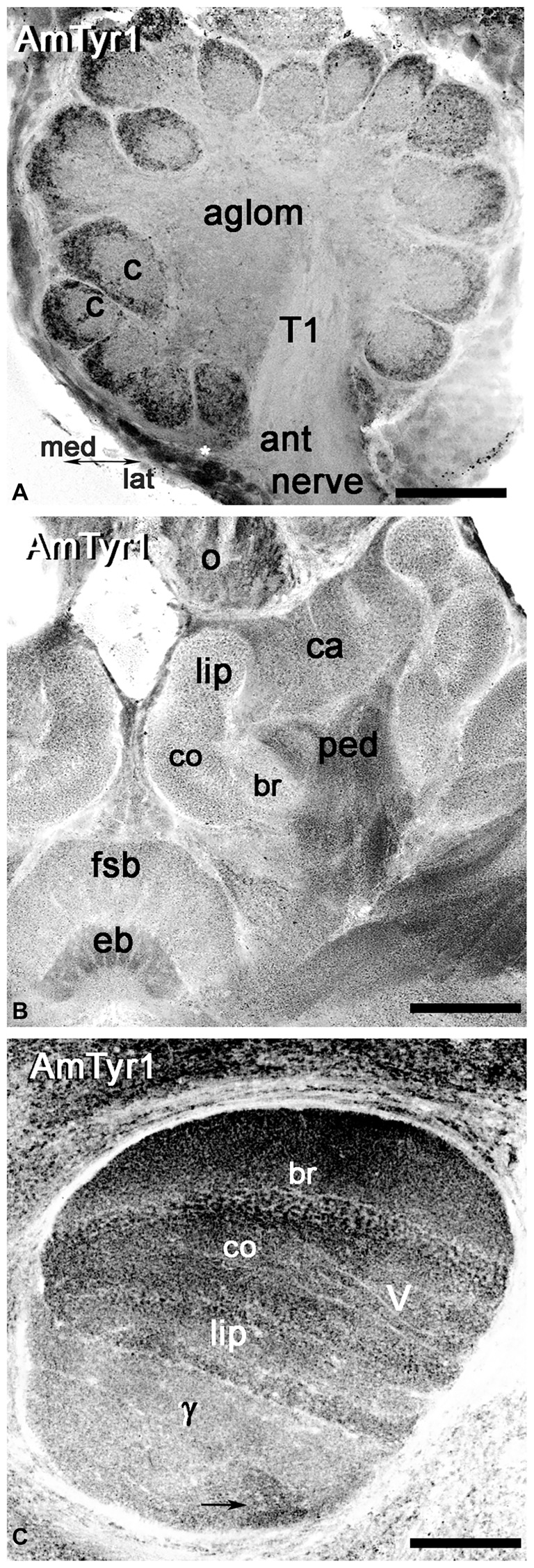
Anti-AmTyr1 labeled the neuropil in the honey bee brain. **(A)** In the antennal lobe, the anti-AmTyr1 is in the cortex area of each glomerulus, but not in the glomerular core (c) and not in the aglomerular neuropil (aglom). The anti-AmTyr1 staining is also absent in the antennal nerve (ant nerve) and olfactory neuron axons tract T1. Asterisk shows a subset of the AmTyr1 positive medial group cell bodies. **(B)** All area of mushroom body calyx (ca) and pedunculus (ped) were labeled with anti-AmTyr1 with various level of intensity. There is a higher density of staining in the pedunculus (ped) of the mushroom body compared to the lip, collar (co) and basal ring (br) area of the calyx. Note: the central complex has anti-AmTyr1 staining in the fan shaped body and ellipsoid body (eb). **(C)** The mushroom body vertical lobe (V) exhibits high-intensity level anti-AmTyr1 staining in a basal ring (br), collar (co) and lip area of the Kenyon cells (KCs) axons that have dendrites in the corresponding area of a calyx. The illustrations in **(A–C)** are inverted fluorescence images. γ—gamma lobe of mushroom body, o-ocelli. Scale bar: **A** = 50 μm, **B** = 75 μm, **C** = 50 μm.

We used anti-AmTyr1 antibodies to characterize the distribution of AmTyr1 in the antennal lobe and mushroom bodies (Figure 4). First, we performed single immunofluorescence staining in unknown age forager bee brains to identify the areas in the antennal lobe and mushroom bodies that labeled with anti-AmTyr1 antibodies (Figure 4). In the antennal lobe, anti-AmTyr1 staining is unevenly distributed within each glomerulus as well in the aglomerular neuropil (Figure 4A). High intensity anti-AmTyr1 staining is localized in the cortex area in each glomerulus, with low intensity staining in the core of the glomerulus and in the aglomerular neuropil (Figure 4A). Anti-AmTyr1 staining is absent in the antennal nerve and tracts in the antennal lobe (only tract T1 is shown in Figure 4A). Cell bodies surrounding the antennal lobe also labeled with different levels of intensity: high intensity labeling is in a subset of the medial group of cell bodies (asterisk) in Figure 4A. In contrast, the lateral group of cell bodies is not positive for anti-AmTyr1. Figure 4B illustrates a section through the mushroom body and central complex. Anti-AmTyr1 immunostaining is present in all mushroom body neuropils: calyx, peduncle, and lobes (Figure 4B). The peduncle and lobes have strong anti-AmTyr1 staining compared with KC bodies and calyx (Figure 4B). The anti-AmTyr1 labeling in the calyx and lobes is particularly distinct in the lip, collar and basal ring areas (Figure 4C). The gamma lobe of the mushroom bodies (staining is shown by the arrow in Figure 4C) had the most variable anti-AmTyr1 immunostaining distribution. Note that the ellipsoid body and ocelli also exhibited a high level of anti-AmTyr1 immunolabeling (Figure 4B).

### ORN Axons Express AmTyr1 in the Antennal Lobe Terminals

**Figure 5:**
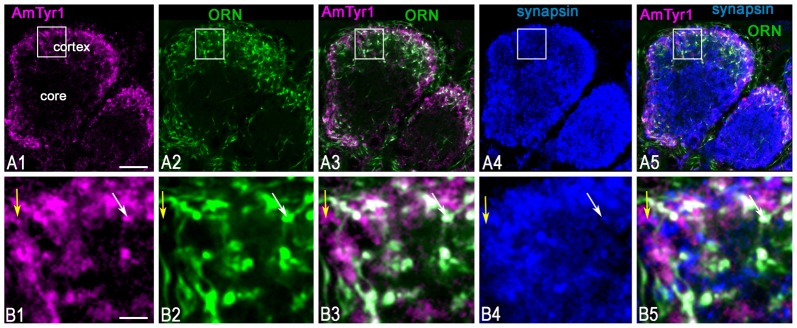
Anti-AmTyr1 labeled synapses of the olfactory receptor neuron (ORN) axons in the antennal lobe glomerulus. **(A,B)** Triple immunofluorescence labeled with anti-AmTyr1 antibodies (magenta), neurobiotin tracer in ORNs (green) and anti-synapsin (blue). **(B)** Images are higher magnifications of details from corresponding squares indicated in **(A)**. **(A1,B1)** Anti-AmTyr1 immunostaining expressed in the cortex of glomeruli (magenta). **(A2,B2)** The ending of the (ORNs, green) revealed by neurobiotin injections into antenna. Anti-AmTyr1 in glomeruli (**A1,A3,A5,B1,B3,B5**, magenta) is in ORN endings (**A2,A3,A5,B2,B3,B5**, green) co-labeled with anti-synapsin (blue, **A4,A5,B4,B5**). The white color in merged images **(A3,A5,B3,B5)** revealed anti-AmTyr1 co-stained in the ORN together with synapsin. The white arrows in **(B1–B5)** indicate co-localization with ORN endings by both anti-AmTyr1 and anti-synapsin; yellow arrow shows co-localization anti-AmTyr1 with synapsin but not with neurobiotin. Scale bar: **A** = 10 μm; **B** = 2 μm.

**Figure 6:**
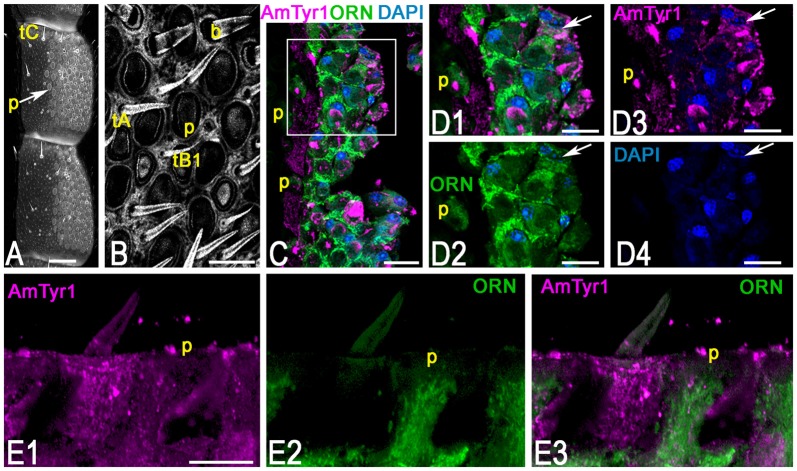
Anti-AmTyr1 immunostaining in the antennal nerve. **(A)** General view of the antennal segments 5 and 6, neurobiotin was injected in the antennal lobe, and the image was obtained by overexposure with the confocal gain to illustrate different types of sensilla (tC–tricoid sensilla type C; Arrow indicates sensilla placodea (p). **(B)** Details of the ventral area of the antenna at higher magnification (tA, tB1, tricoid sensilla type A and B1 respectively, b-basiconic sensilla). **(C)** The section via antenna illustrates merged images of the group of ORN cell bodies and various processes labeled with neurobiotin tracer (green) anti-AmTyr1 (magenta), and 4′,6-diamidino-2-pheylindole (DAPI), marking the nucleus, P-indicate the fibers in the sensilla placodea. **(D)** Images present higher magnifications of details from corresonding squares indicated in **(C)**. **(D1)** shows of cell bodies and processes labeled with neurobiotin (green) and anti-AmTyr1 staining (magenta, single staining) and nuclei (blue, DAPI). **(D2)** illustrates only neurobiotin labeled processes (green) and nuclei (DAPI, blue). **(D3)** illustrates only anti-AmTyr1 (magenta) and nuclei (DAPI). **(D4)** shows the nuclei staining. The arrow indicates cell bodies that have co-staining with AmTyr1 and neurobiotin. **(E)** Anti-AmTyr1 (**E1** single image, magenta) is in the area of sensilla placodea (p) with dendrites of ORNs labeled with neurobiotin (green **E2**). The absence of the white staining in merged image **(E3)** demonstrates that AmTyr1 does not co-label dendrites of labeled ORNs. Scale bar: **A** = 100 μm; **B,E** = 20 μm; **C** = 25 μm; **D** = 10 μm.

To identify the primary cell types that express AmTyr1 in the antennal lobe we used triple immunofluorescence staining. The images in Figures 5A1–A5, show triple immunofluorescence staining in glomeruli with anti-AmTyr1 (magenta, Figure 5A1), neurobiotin backfills of antennal axons (green, Figure 5A2) and anti-synapsin (blue, Figure 5A4). Close-ups of ORN endings in the box in Figures 5A1–A5 is illustrated in Figures 5B1–B5, respectively. The distribution of anti-AmTyr1 is in the cortex of the glomerulus (magenta, Figures 5A1,B1), where the ORN terminals are located (green, Figures 5A2,B2). The endings of ORNs represent varicosities that synapse on the neuronal processes located in the cortex of the glomerulus. In the merged image (magenta anti-AmTyr1 and green ORNs, Figures 5A3,B3), the white color demonstrates co-labeling of anti-AmTyr1 in the ORN axon endings (Figures 5A3,B3). Therefore, ORNs containing neurobiotin also have anti-AmTyr1 staining (Figures 5A3,B3). Anti-synapsin also co-labeled the neurobiotin containing ORNs (Figures 5A4,B5). An example of staining with anti-AmTyr1 and anti-synapsin in ORNs is shown by the white arrows in Figures 5B1–B5. However, anti-AmTyr1 labeled processes other than ORN axons. The yellow arrows in Figure 5B1 demonstrate co-staining of anti-AmTyr1 with anti-synapsin but not with neurobiotin. These data suggest that other cell types could express AmTyr1 in the cortex of glomeruli, or possibly not all ORNs were labeled with neurobiotin in our preparations. Also, due to the limitation of light microscopy we cannot exclude that AmTyr1 is expressed in processes of the glomerular cortex that are not co-labeled with anti-synapsin. More work needs to be done to identify all possible antennal lobe neurons and/or glial cells expressing AmTyr1. To reveal ORN cell bodies and dendrites in sensilla, we injected neurobiotin in the antennal lobe (Figure 6). Then the antenna was opened and processed for double immunofluorescence staining to reveal neurobiotin (green) and AmTyr1 (magenta). The neurobiotin labeled ORN cell bodies and processes in sensilla (Figure 6). Figures 6A,B illustrates a frontal view of antennal subsegments 5 and 6 of a flagellum. This antenna was not cut during immuno-procedures, and the images were taken in a confocal mode with overexposure of all laser channels for illustration of the surface area of an antenna with different types of sensilla. The three olfactory sensilla are identified as sensilla placodea (p in Figures 6A,B), sensilla basiconica (b, Figure 6B), sensilla trichodea type A (tA, Figure 6B) and type B1 (tB1, Figure 6B), sensilla trichodea type C (Figure 6A, tC). In the honey bee, sensilla placodea (or poreplate) house between 7 and 30 ORNs (Esslen and Kaissling, 1976). To reveal anti-AmTyr1 and neurobiotin simultaneously in the antennal processes, antennae were cut with a razor into two halves and each half processed for staining with anti-AmTyr1, neurobiotin and DAPI; the latter is the marker for cell nuclei (Figure 6C). Neurobiotin processes in the sensilla placodea are not co-labeled with anti-AmTyr1. However, the neurobiotin filled ORN cell bodies (green Figures 6C,D) co-labeled with anti AmTyr1 (magenta in Figures 6C,D). The example of a cell with co-localization with AmTyr1 is indicated by the arrow in Figure 6D. The anti-AmTyr1 labeled processes in the sensillum lymph area (Figure 6E1), but they are not co-localized with neurobiotin labeled dendrites in the sensilla placodea (Figure 6E2) as demonstrated by the absence of white color in the merged image (Figure 6E3).

### The uPN Terminals in the Calyx of the Mushroom Body Express AmTyr1

The calyx of the mushroom bodies consists of intrinsic neuron (KC) dendrites and afferent neuronal terminals of uniglomerular PNs (uPNs) from the antennal lobe (lip and basal ring), gustatory inputs (area between lip and collar) and visual inputs (collar). Interestingly, the gustatory inputs to mushroom body calyx are from subesophageal neurons, the same brain region where the modulatory octopaminergic/tyraminergic VUM neurons originate. In addition, other modulatory neurons and GABAergic inputs are present in the calycal neuropil. The organization of the calyx is microglomerular, which reflects interactions of uPN axons (input) with dendrites of KCs (output) together with the inputs of inhibitory (GABA) and modulatory afferent neurons.

**Figure 7:**
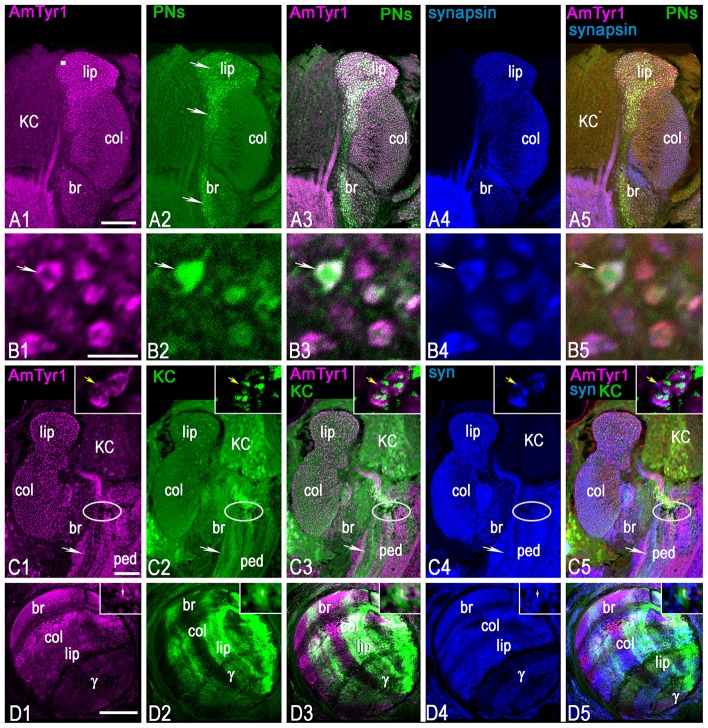
Triple staining with anti-AmTyr1 (magenta) and anti-synapsin (blue) in the mushroom body after neurobiotin injection in uniglomerular projection neurons (uPNs; green, **A,B**) and subsets of KCs (green, **C,D**). **(A)** Anti-AmTyr1 antibodies (magenta, **A1**) co-label the neurobiotin injected uPNs ending in the calyx (green, **A2**). Arrows in **(A2)** show the axon from uPNs entering the basal ring and lip area of the calyx and labeling presynaptic parts of microglomeruli. Images in **(B)** illustrate at higher magnification microglomeruli indicated by the square in **(A1)**. Arrows in **(B)** show that single microglomeruli label with anti-AmTyr1 (magenta, **B1**) in a uPN terminal bouton **(B2)**. The white staining in merged images **(A3,B3)** indicates co-labeling of anti-AmTyr1 with uPN terminal microglomeruli. The microglomeruli labeled with anti-synapsin as a presynaptic marker (**A4,B4**, blue). The white staining in merged triple staining image **(A5,B5)** indicates that anti-AmTyr1 and anti-synapsin are co-labeled in uPN microglomeruli. **(C,D)** Anti-AmTyr1 labeled microglomeruli (magenta, **C1**, insert in **C1**) in the calyx, subsets of KC bodies, the pedunculus (ped, **C1**). Also, areas of the mushroom body vertical lobe that correspond to KCs with dendrites in the basal ring, lip and collar areas of the calyx have a high level of staining intensity **(D1)**. The (KCs) were injected with neurobiotin in the area indicated in **(C)** by an ellipse, and in single image staining (green, in **C2,D2**) the neurobiotin revealed in cell bodies, dendrite in calyx (**C2**, insert in **C2**) and in lobe **(D2)**. Only subsets of KCs took up the neurobiotin in this preparation. For the injection of neurobiotin in the area shown by the ellipse in **(C)**, the subsets of KCs that took up the tracer express it in cell bodies, in dendrites in the lip, basal ring and collar (**C2**, insert in **C2**), and in the axons of the corresponding area of vertical and gamma lobe **(D2)**. Merged images **(C3,D3)** show the co-localization of KCs that take up neurobiotin with AmTyr1 in axons but not in the dendrites of the calyx (inserts in **C3**). **(C)** AmTyr1 (magenta **C1**) expression in subsets of mushroom body KC axons (green, **C2**) but not in the dendrites in the calyx (insert in **C**). The merged images in **(C5)** illustrate co-localization of anti-AmTyr1 with anti-synapsin (blue, **C4** single staining) in calyx microglomeruli, but not with neurobiotin labeled KC dendrites in calyx (insert in **C4**,**C5** respectively). Anti-AmTyr1 in the mushroom body lobe is in KC axons; these KCs have dendrites in the basal ring and collar areas. In insert **(D1)**-AmTyr1 (magenta) is in axons of KC labeled with neurobiotin (**D2**, single image) and co-labeled with synapsin in (**D4**; single image). In the triple staining image (**D5**; insert **D5**) the white color corresponds to co-labeling of synapsin in KC axons and AmTyr1. Note, that not all synapsin labeling processes (single staining image **D4**, insert **D4**) express AmTyr1 (single staining image **D1**, insert **D1**). Arrows in insert **(D1–D5)** shows AmTyr1 in the axon of KC co-labeled with synapsin. Scale bar: **A,C,D** = 50 μm, **B** = 2 μm.

Anti-AmTyr1 antibodies labeled microglomeruli in the calyx of the mushroom bodies (Figure 7A1). The higher magnification in image (Figure 7B1) represents the equivalent of an area shown in the square box in Figure 7A1 and illustrates a few of the microglomeruli. The anti-AmTyr1 is in the cortex of the microglomeruli, but the core is free from immunostaining.

To mark the neuronal structure of uPN terminals in the calyx microglomeruli, neurobiotin was injected in the antennal lobe to reveal uPN boutons in the basal ring and lip areas of the mushroom body calyx (arrows in Figures 7A2,B2). The dye revealed uPN boutons (Figure 7B2), which are mainly presynaptic to KC dendrites. As demonstrated in Figures 7A3,B3, anti-AmTyr1 co-labeled with uPN boutons and was localized in the periphery of the bouton (white color on merged images of Figures 7A1,A2,B1,B2). Anti-synapsin labeled the presynaptic sites of the microglomeruli (Figures 7A4,B4). When the images (anti-synapsin, anti-AmTyr1 and uPNs terminals) are merged in Figures 7A5,B5, the white color demonstrates co-expression of anti-AmTyr1 and synapsin in the periphery of microglomeruli within the same boutons as uPNs. Thus, AmTyr1 is localized in presynaptic sites of uPNs in microglomeruli of mushroom body calyx.

### A Subset of Kenyon Cells in the Basal Ring, Lip and Collar Express AmTyr1 in Cell Bodies and Axons but Not in the Dendrites in the Calyx

The mushroom body KC bodies express a low level of AmTyr1 staining compared to staining in the calyx (Figure 7C1). The anti-AmTyr1 marks KC bodies in the basal ring, lip and collar and scattered cell bodies of the gamma lobe with very low intensity, although it is possible to distinguish a different level of staining in subsets of cells with higher expression in the basal ring KCs (Figure 7C1). The anti-AmTyr1 staining is strong in the vertical mushroom body lobe, especially in the areas that receive axons from KCs in the basal ring, the collar and the lip areas. In comparison, staining is much lower in the gamma lobe (Figure 7D1).

KCs are the intrinsic neurons that make up the mushroom body. They have dendrites in the calyx and axons in the lobe, and different types of afferent (input/output) neurons make their connection to the KCs in the calyx and lobe. To identify localization of the AmTyr1 receptor in KC dendrites and axons, we labeled KCs with neurobiotin before immunostaining procedures with anti-AmTyr1 (Figures 7C2,C3,D2,D3) and anti-synapsin (Figures 7C4,C5,D4,D5) antibodies. For neurobiotin injected in the calyx of the cell body layer (Figure 7C), the area of injection was marked by an ellipsoid in the peduncle of the mushroom body. The dye was taken up by subsets of KC bodies and their dendrites in the calyx as well in the axons in the peduncle (Figure 7C2) and lobes (Figure 7D2).

The neurobiotin-injected KC dendrites in the calyx were not labeled with anti-AmTyr1. The insert in Figures 7C1,C5 shows typical AmTyr1 staining in the microglomeruli. In the insert in Figure 7C2, fine green fibers of KCs are in close proximity to the AmTyr1 stained PNs, but there is no co-labeling as demonstrated in Figure 7C3 and in the insert, where the white color expected for co-labeling in the dendritic area of KCs is absent. The AmTyr1 receptor is co-expressed in the bundles of axons of neurobiotin labeled KC in the pedunculus (white arrow in Figures 7C2,C3). The subsets of neurobiotin labeled KCs (green bundles in Figures 7C2,C3,C5) could be traced to the mushroom body basal ring, the collar, the lip and gamma lobe areas (green in Figures 7D2,D3,D5). KC axons co-express AmTyr1 in the basal ring, collar and lip area of the mushroom body (Figure 7D3). The insert of Figures 7D1,D2 show the area of the lip KC axons in the vertical lobe, where only a few fibers express receptors as indicated by the yellow arrows in inserts. The Figures 7C4,D4,C5,D5 show anti-synapsin staining in the calyx and lobes and anti-synapsin co-labeled with KCs axons in the pedunculus and lobe, with subsets of KCs that co-express AmTyr1 in the same axons.

## Discussion

Tyramine and octopamine play important roles in insect behavior by acting as neurotransmitters, neurohormones and neuromodulators (Roeder, 2005). Their function is analogous to the adrenergic/noradrenergic system in vertebrates. In the honey bee (*Apis mellifera*), the cellular sources of octopamine (Kreissl et al., 1994; Sinakevitch et al., 2005) and the distribution of one receptor—AmOA1—has been previously described in detail (Sinakevitch et al., 2011, 2013). In the current study, we revealed cellular sources for tyramine and the distribution of one TYR—AmTyr1—in the honey bee brain by using immunocytochemistry with tyramine antiserum (Kononenko et al., 2009; Homberg et al., 2013) and a newly characterized anti-AmTyr1 antibody.

### Tyramine-Immunostaining in the Honey Bee Brain

A total of approximately 160 tyramine immunoreactive neurons are organized in different clusters in the brain. They are the source of tyraminergic fibers with small varicosities in the optic lobes, antennal lobes, lateral protocerebrum, mushroom body calyces and gamma lobes, tritocerebrum and SEG. Also, tyramine-like immunoreactive fibers are present in the antennal nerve and in the nerve innervating the corpora cardiaca (NCC1). Since tyramine is a precursor of octopamine, it is not surprising that our studies revealed that tyramine immunostaining is largely in the same clusters of cells as octopamine, as previously described in the honey bee brain (Kreissl et al., 1994; Sinakevitch et al., 2005). We summarized the cell numbers in Table 1 and used the same nomenclature of cell groups as Sinakevitch et al. (2005). According to our present report and comparison with previous studies on octopamine immunoreactivity in the honey bee (Kreissl et al., 1994; Sinakevitch et al., 2005), tyramine containing neurons described here are largely the same as those that contain octopamine due to the position of their clusters.

We also found differences from earlier studies (Sinakevitch et al., 2005), as reflected in Table 1. For example, we did not find tyramine immunoreactive cells in octopamine-positive clusters G0b, G1 and G5a. It could be that those cells convert tyramine to octopamine rapidly, such that the tyramine titers are below detection levels. We also found that there is a higher total number of cells containing tyramine immunoreactivity in comparison to octopamine immunoreactive cells reported by Kreissl et al. (1994) and Sinakevitch et al. (2005). Previous work on biogenic amine immunostaining reported that handling procedures could significantly alter octopamine/tyramine levels, and possibly make one or both undetectable by immunostaining (Sinakevitch et al., 2005; Kononenko et al., 2009; Homberg et al., 2013). However, the differences in numbers of octopaminergic and tyraminergic neurons in the honey bee brain might also be due to the presence of neurons that are only tyraminergic. Therefore, due to the higher number of tyramine-containing cells, and to a large amount of tyramine-containing profiles in neuropils, our data suggest that some cells could release only tyramine, some cells either release only octopamine or co-release both biogenic amines.

### Tyramine Immunoreactivity in the Antennal Nerve

We also found that the antennal nerve contains tyramine-like immunoreactivity originating from processes in SEG neurons that enter the antennal nerve via T5-6. However, it is not clear what neurons in the SEG could be the source of these fibers: e.g., the neurons from cluster six or unidentified median neurons from mandibular and maxillary neuromeres. Tyramine in the antenna could be in the axons of neurons innervating the head and antennal muscles, and it could also act as a hormonal release in the antenna to modulate antennal sensory neurons. Furthermore, tyramine in the antennal lobe could also be involved in the regulation and modulation of sucrose responsiveness (Scheiner et al., 2002). It seems clear, however, that tyramine does not modulate ORN cell bodies via AmTyr1, because we did not observe AmTyr1 on the cell bodies of ORNs.

### Tyramine Immunoreactivity in the Antennal Lobe and Mushroom Body

The primary sources of tyramine in the antennal lobe and calyx of the mushroom body are from at least two median neurons with cell bodies in the SEG: VUMmd and VUMmx neurons. In the antennal lobe, these neurons innervate the cortex of each glomerulus, where ORN axons terminate. They also branch to the LH and calyx of the mushroom body via the lateral antenno-protocerebral tract. The entire protocerebral neuropil, except for the mushroom body lobes, is penetrated by very fine varicosities of tyramine-containing fibers, and their sources are the VUM neurons from the SEG as well as the cells from clusters G2 and G3. The mushroom body lobe has very few tyramine containing fibers, only a few branches in the gamma lobe are clearly visible, and the source could be the laterally paired neurons from the SEG (Schröter et al., 2007).

Also, the presence of tyramine in NCC1 could indicate that the corpora cardiaca could receive tyramine-containing branches from some VUM neurons located in the SEG. The morphology of VUM neurons that give rise to axons in the NCC1 was previously described in the labial and maxillary neuromeres by Eichmüller et al. (1991). While the role of tyramine in NCC1 is unclear, it could be that tyramine is released from these neurons to act as a neurohormone, or/and it could control the release of other hormones from the corpora cardiaca.

### AmTyr1 Receptor Structure

Activation of AmTyr1 in heterologous expression systems leads to reduction of cAMP (Blenau et al., 2000; Reim et al., 2017). Both tyramine and octopamine reduce cAMP when bound to AmTyr1 (Blenau and Baumann, 2016), but tyramine is more potent than octopamine. Moreover, AmTyr1 binds yohimbine, which is an antagonist of the AmTyr1 receptor (Blenau and Baumann, 2001, 2016; Reim et al., 2017). In our 3D model, AmTyr1 has a long intracellular loop 3 and a short C-terminus. Those properties are similar to other GPCRs linked to inhibition of adenylyl cyclase activity; for example, the alpha 2-adrenergic receptors also have a long intracellular loop 3 and a short C-terminal tails (Kuhar et al., 1999; Rosenbaum et al., 2009). Based on the structural and pharmacological properties, AmTyr1 is similar to the vertebrate type alpha 2-adrenergic receptors, which when activated also reduce cAMP, and yohimbine has also high affinity of this vertebrate receptor type. The function of vertebrate-type alpha 2-adrenergic receptors is primarily for inhibitory presynaptic control of the release of norepinephrine, ATP and acetylcholine from the nerve (Rosenbaum et al., 2009).

### AmTyr1 Receptors Are Expressed in the Presynaptic Sites of ORN and uPN Axons

The anti-AmTyr1 antibodies we used specifically recognize the AmTyr1 protein, and staining mapped with high intensity to honey bee brain neuropil areas. Some cell bodies immunolabeled with low intensity compared to the neuropil, which could reflect that the AmTyr1 receptor was translated in the cell body and transported to axonal terminals. The present localization studies on anti-AmTyr1 receptor protein distribution provide a further confirmation of the previous work of Mustard et al. (2005), where *in situ* hybridization of the AmTyr1 mRNA was reported to be expressed in the mushroom body and antennal lobe neurons.

**Figure 8:**
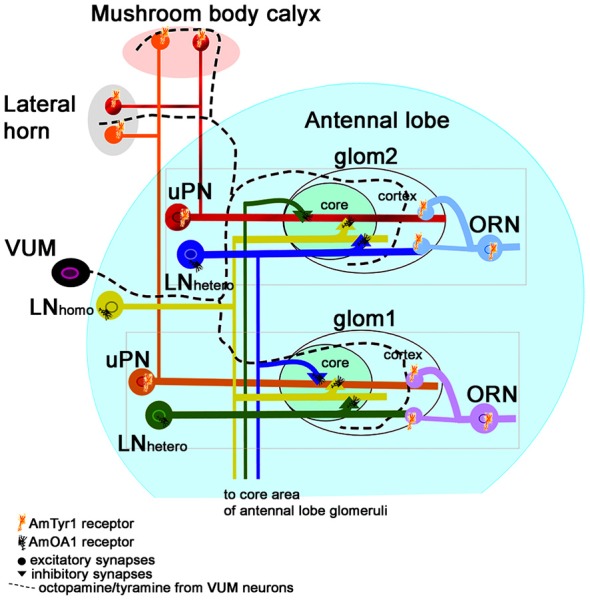
Schematic view of the neural network proposed for the honey bee antennal lobe (modified from Sinakevitch et al., 2013). Each glomerulus can be defined by three types of neurons that are tuned to a narrow range of odorants: (i) ORN axons that project excitatory branches into the cortex of the glomerulus; (ii) the glomerular uPNs that receive input in both the cortex and core of the glomerulus and project excitatory output branches to the LH and Mushroom body calyx; and (iii) inhibitory hetero-LNs that branch in all areas of the glomerulus (cortex and core), where they receive excitatory output from the cortex and inhibitory from the core. Hetero-LNs also have inhibitory input in the core area of one glomerulus. Hetero-LNs also have two types of neurotransmitter (GABA and Histamine, Dacks et al., 2010). The neurons that interconnect all glomeruli are multiglomerular LNs (containing both GABA and Allatostatin, Kreissl et al., 2010). They have input/output branches in the core area where they inhibit neurons in the core. There are also multiglomerular inhibitory GABAergic mPNs that are not illustrated here. We propose that VUM neurons release both octopamine and tyramine in the antennal lobe, LH and mushroom body calyx. Each glomerulus will respond to the presence of each biogenic amine through specific receptors. Inhibitory LNs (hetero and homo) express AmOA1. The ORN axons express AmTyr1, and the uPNs axons in the LH and Mushroom body calyx also express AmTyr1 receptors. In both cases, AmTyr1 is in a position to regulate excitatory transmission into the respective areas. We hypothesize that the action of octopamine and tyramine released by VUM could be dependent on the ratio of the amines and on the specific target cells that express the receptors. An excess of octopamine in a glomerulus leads to inhibiting the inhibition in the core and simultaneously blocks excitation in neighboring glomeruli via AmOA1 on GABAergic LNs. An excess of tyramine inhibits the release of the excitatory neurotransmitter in the synapses.

Anti-AmTyr1 immunolocalization studies revealed that in the antennal lobe AmTyr1 is expressed in the presynaptic sites of ORN axons as they innervate the cortex of glomeruli. Similarly, in the mushroom body calyx, AmTyr1 is expressed in the presynaptic sites of uPN axons located primarily in the microglomeruli of the lip and basal ring calyx areas. AmTyr1 is expressed in areas innervated by VUM (md and mx) neurons. Therefore, release of tyramine from VUM (md and mx) neurons in the antennal lobe and mushroom body could target presynaptic sites of ORNs and uPNs (Figure 8). Because the AmTyr1 receptor is similar in structure and function to the vertebrate alpha-adrenergic receptor type 2 (Kuhar et al., 1999), we hypothesize that the release of tyramine from tyramine containing VUM neurons could inhibit excitatory neurotransmitter release in the presynaptic axons of ORNs and uPNs. The main excitatory neurotransmitter in ORNs and uPNs is acetylcholine, which plays important roles in olfaction and memory in bees (Gauthier, 2010). AmTyr1 in theses neurons might be involved in regulation of the release of acetylcholine. At the same time the release of octopamine from VUM neurons could coordinate excitation via AmOA1 in inhibitory neurons in the antennal lobe and mushroom bodies (Sinakevitch et al., 2013, Figure 8).

### AmTyr1 Receptors Are Expressed in Axons of Kenyon Cells in the Lobe but Not in Dendrites

Only subsets of KCs express the AmTyr1 receptor in the axons and cell bodies, with a higher level of expression in the axons compared to cell bodies. We did not find any detectable staining in the dendrites of KCs located in calyx. In the KCs axons, AmTyr1 co-localized with synapsin, the presynaptic marker of the neurons. In the mushroom body lobe, the anti-AmTyr1 positive subsets of KCs axons are in the pedunculus, lip, collar and basal ring area of the lobe. The branching pattern of the KCs in the mushroom body lobes was described in detail by Strausfeld (2002).

### Tyramine Receptor Distribution in Comparison to Other Insects

Homolog of AmTyr1 have been studied in other insects: fruit fly (TyrR, Oct/TyrR, TYR, Tar1, CG7485; Saudou et al., 1990; El-Kholy et al., 2015); locust (Vanden Broeck et al., 1995); silkworm (Ohta et al., 2003); and cockroach (PeaTyr1, Rotte et al., 2009). Similar to AmTyr1, when investigated in expression systems, these Tyr1 receptors were also negatively coupled to adenylyl cyclase via Gi protein (Uzzan and Dudai, 1982; Aoyama et al., 2001; Blenau and Baumann, 2003). However, activation of locust and fruit fly Tyr1 receptors have also been shown to mobilize intracellular calcium (Ohta and Ozoe, 2014). The brain distribution of PeaTyr1 in the cockroach *Periplaneta americana* is similar to our results with AmTyr1, but that report did not identify specific neurons such as the presynaptic sites we show here (Rotte et al., 2009). Additionally, they reported expression in glial cells and peripherals organs. In our study, we also report the possible expression of AmTyr1 in glial cells and peripheral organs, but we did not illustrate it here. Expression of the moth (*Agrotis ipsilon*) OA/TYR (AipsOAR/TAR) was also reported in the antennae, the antennal lobes and the brain and in the antennal lobes was shown to be regulated with age (Duportets et al., 2010). In the fruit fly *Drosophila melanogaster*, the expression pattern of TyrRs was studied by utilizing the presumptive promotor regions of the TYR and the Gal4/UAS system (El-Kholy et al., 2015). The fruit fly TyrRs are expressed in the tracheal system, in salivary glands and in the mushroom body and ellipsoid body, glial cells, fat body and muscles. However, the fruit fly has three TYRs, one of which does not have an ortholog in the honey bee (Tyr3), which makes it difficult to compare our results on localization with the fruit fly (El-Kholy et al., 2015).

### Tyramine and AmTyr1 Receptors in the Mushroom Body Lobe

It was striking in our study that the mushroom body α- and γ-lobes do not have a high amount of tyraminergic (present studies) or octopaminergic fibers (Sinakevitch et al., 2005) in contrast to the calyx of the mushroom body and the protocerebral area that surrounds mushroom body lobes. However, the mushroom body lobes express both the AmTyr1 (present study) and AmOA1 receptors (Sinakevitch et al., 2011, 2013). We propose that the source of tyramine in the mushroom body lobe could be from the hemolymph or from the release of tyramine in the protocerebrum followed by diffusion into the lobes. The presence of tyramine in the hemolymph was reported in locust, and the level of tyramine is lower than octopamine in brain tissue as well in the hemolymph.

It may be important for behavioral studies to consider the ratio of tyramine and octopamine in the tissue rather than focusing exclusively on a single amine, since they have potentially antagonistic effects (Roeder, 2005). Fussnecker et al. (2006) showed that honey bee flight behavior was affected inversely by octopamine and tyramine treatment. Octopamine increased flight behavior, while tyramine treatment decreased it. Fussnecker et al. (2006) suggested that both biogenic amines affect central pattern generators or interact with sensory perception. Tyramine and octopamine have opposite effects on locomotion in the fruit fly (Saraswati et al., 2004), and have been shown (Brembs et al., 2007) to reduce fruit fly flight initiation. Tyramine also reduces the stimulatory effect of octopamine in the fly (Uzzan and Dudai, 1982). In the honey bee brain tyramine is involved in habituation of an appetitive reflex (Braun and Bicker, 1992) and in inhibition of the initiation of foraging behaviors (Schulz and Robinson, 2001). High brain tyramine in queenless honey bee workers might inhibit foraging behavior and encourage them to stay in the nest and become reproductive workers (Sasaki and Nagao, 2002).

## Conclusion

One of the important findings in our studies is that tyramine could originate from VUMmx and VUMmd neurons in the antennal lobe, lateral protocerebrum and mushroom body calyx. Our present tyramine mapping results in the honey bee are consistent with reports on octopamine/tyramine containing cells in locust (Kononenko et al., 2009; Homberg et al., 2013) and fruit fly (Monastirioti et al., 1996; Monastirioti, 1999; Sinakevitch and Strausfeld, 2006; Busch et al., 2009; Busch and Tanimoto, 2010; Selcho et al., 2014). Our findings suggest that tyramine is not only the precursor of octopamine but could also be an independent neurotransmitter. From the AmTyr1 distribution, tyramine targets the excitatory synapses of ORNs in glomeruli and of PNs in the calyces of the mushroom bodies. From the similarity to the vertebrate alpha 2 type adrenergic receptor, we suggest that tyramine could inhibit release of the neurotransmitter from both ORNs and PNs. Since the same neurons (VUMmd and mx) have both tyramine and octopamine, it is possible that the ratio of tyramine/octopamine in proximity to the receptors plays a crucial role in the physiological responses of the cells. Also, tyramine and octopamine could be released in hemolymph, which brings additional complex response in brain circuits as the hemolymph circulates through the brain.

## Author Note

During revision of our manuscript a new article about tyramine and the AmTyr1 receptor (in this article the authors call it “AmTAR1”) was published (Thamm et al., 2017). The authors developed new antibodies against AmTyr1 raised against a cytoplasmic domain of the receptor, whereas our antibodies targeted an extracellular domain. They described staining in sections of the entire brain including the olfactory neuropils, which was the exclusive focus of our work. The two studies show similar results in regard to the receptor in the mushroom bodies but differ in regard to antennal lobe staining. In our anti-tyramine staining, we used the same anti-tyramine antibodies as Thamm et al. (2017), but we used a different staining procedure. There were differences between the studies in the number of tyraminergic cells and location of tyramine in the mushroom body lobes. These differences in tyramine and AmTyr1 receptor expression will require further study.

## Author Contributions

ITS and BHS designed experiments and wrote the manuscript, SMD made a western blot, the bioinformatic analyses of the AmTyr1 receptor, ITS executed all experiments and made the illustrations.

## Conflict of Interest Statement

The authors declare that the research was conducted in the absence of any commercial or financial relationships that could be construed as a potential conflict of interest.
